# Influenza A Virus Detected in Native Bivalves in Waterfowl Habitat of the Delmarva Peninsula, USA

**DOI:** 10.3390/microorganisms7090334

**Published:** 2019-09-09

**Authors:** Christine L. Densmore, Deborah D. Iwanowicz, Shawn M. McLaughlin, Christopher A. Ottinger, Jason E. Spires, Luke R. Iwanowicz

**Affiliations:** 1 U.S. Geological Survey, Leetown Science Center, 11649 Leetown Road, Kearneysville, WV 25430, USA; 2 National Oceanic and Atmospheric Administration, Cooperative Oxford Laboratory, 904 South Morris Street, Oxford 21654, UK

**Keywords:** avian influenza virus, AIV, bivalve, mollusk, clam, mussel, Delmarva Peninsula, Chesapeake Bay, avian, waterfowl disease

## Abstract

We evaluated the prevalence of influenza A virus (IAV) in different species of bivalves inhabiting natural water bodies in waterfowl habitat along the Delmarva Peninsula and Chesapeake Bay in eastern Maryland. Bivalve tissue from clam and mussel specimens (*Macoma balthica*, *Macoma phenax*, *Mulinia* sp., *Rangia* *cuneata*, *Mya* *arenaria*, *Guekensia demissa*, and an undetermined mussel species) from five collection sites was analyzed for the presence of type A influenza virus by qPCR targeting the matrix gene. Of the 300 tissue samples analyzed, 13 samples (4.3%) tested positive for presence of influenza virus A matrix gene. To our knowledge, this is the first report of detection of IAV in the tissue of any bivalve mollusk from a natural water body.

## 1. Introduction

Bivalve mollusks provide invaluable benefits to their aquatic environments. As suspension feeders, bivalves such as clams, mussels, and oysters filter particulate matter from bay water and are widely regarded as a water quality control mechanism in the Chesapeake Bay ecosystem [1,2]. Filtration of bay water by Eastern oysters (*Crassostrea virginica*) alone is substantial; typical filtration rates are approximated as 5 L water/h/g dry tissue during peak filtration periods in summer [3], translating to over 120 L of water filtered each day by a single market sized oyster. Other bivalves such as the clams and mussels that inhabit Chesapeake Bay and its tributaries also represent substantial biomass in the region and contribute substantially to water filtration rates. Biomass of the non-native clam *Rangia cuneata* has been reported to range from 7.7 to 29 g C m^−2^ throughout fresh and brackish water up to approximately 10 ppt salinity in Chesapeake Bay and some of its major tributaries, and even the small Baltic clam (*Macoma balthica*) has comparable biomass levels in the region [4]. Chesapeake bivalves may also bioaccumulate microorganisms in the process of water filtration and feeding. Eastern oysters have demonstrated capacity to accumulate protozoal pathogens such as *Cryptosporidium parvum* and *Giardia* sp., bacteria such as *Vibrio* sp. and fecal coliforms, and enteric viral agents such as Hepatitis A virus [5,6,7]. Other bivalve species may similarly bioconcentrate pathogens of importance to wildlife and human health interests. For instance, zebra mussels (*Dreissena polymorpha*) from the Shannon River drainage in Ireland were found to carry *C. parvum* oocysts, *Giardia lamblia* cysts, *Encephalitozoon intestinalis* spores, and *Enterocytozoan beineusi* spores [8]. In the Chesapeake Bay region, *Giardia* sp. cysts have also been recovered from *Macoma* spp. clams in the Rhode River subestuary [9].

Type A influenza viruses (IAV) are infectious to many species of wild and domestic birds as well as many mammals. With multiple viral subtypes that vary considerably in pathogenicity among hosts, the environmental distribution and longevity of IAV is an important consideration for wildlife, domestic animal, and human health interests. Persistence of IAV in pond sediment from Alaskan waterfowl habitat has been demonstrated with a virus detection rate greater than 50% among samples [10]. In waterfowl habitat on the Delmarva Peninsula, virus detection rates of up to 25% have been observed from sediment collected from freshwater impoundments [11]. Laboratory experiments have demonstrated that these viruses can persist in water for up to several months with suitable water conditions, including pH, salinity, and cold temperatures [12]. Furthermore, IAV has been isolated from potential biotic reservoirs in the aquatic environment. Bioconcentration of IAV has been demonstrated among filter-feeding bivalves including zebra mussels (*D. polymorpha*) and Asian clams (*Corbicula fluminea*) under laboratory conditions [13,14].

Building upon this previously demonstrated premise that filter-feeding bivalves may bioconcentrate IAV in a controlled setting, the purpose of this study was to evaluate the capacity of native bivalves to accumulate detectable levels of IAV within a natural water body in waterfowl habitat in a region where large numbers of waterfowl concentrate during migration and overwinter. The coastal Delmarva Peninsula in Maryland is part of the Chesapeake Bay catchment and serves as an ideal location for this study, as it provides habitat for both a variety of bivalves in fresh, brackish, and saltwater and a high density of overwintering migratory waterfowl with a proven capacity to carry IAV.

## 2. Results

Of the 300 tissue samples analyzed, 13 (4.3%) were positive for the IAV matrix gene. Positive samples represented 12 individual mollusks (12/264 or 4.5%) including six of the 24 *G. demissa* mussels (25%) from Reed Creek, four of the 40 (10%) *M. phenax* clams sampled from Goldsborough Creek, and two of the 59 (3.4%) *M. balthica* clam specimens from Trippe Creek (Table 1). As previously described, there is considerable precedent for isolation of pathogenic microbes from bivalve mollusks in natural waters, but to our knowledge, this is the first report of detection of an avian influenza virus in the tissue of any bivalve mollusk collected from a natural water body.

## 3. Discussion

These bivalve specimens testing positive for IAV represented three different species including *Macoma* spp. clams and mussels. The *Macoma* species are widespread and abundant in sediment of shallow waters throughout the Chesapeake Bay and its tributaries, acting as both suspension feeders and deposit feeders [2,15]. *Guekensia demissa*, the positive mussel specimen, is a suspension feeder in the intertidal zone in Chesapeake Bay and its tributaries [16]. The positive specimens were also obtained from each of the three creek sites in this study but not from the locations near the mouth of the Chester River (Eastern Neck) and the open bay (Barren Island) despite the latter two locations being part of the Chesapeake Marshlands National Wildlife Refuge Complex and recognized as areas for congregation of large numbers of waterfowl or shorebirds. All sites sampled in this study were within the mesohaline portions of the Chesapeake Bay catchment, and the sites yielding the IAV- positive specimens were within regions with mean surface salinity of approximately 7.6–12.5 ppt [17]. Water temperatures at the time of specimen collections were in the 15–17 °C range and water pH was slightly basic (7.5–7.8). These parameters are consistent with reported water quality values that support presence and infectivity of IAV in water, as these viruses are most stable in water below 17 °C in pH ranges of 7.4–8.2 and at salinity under 20 ppt [12].

Avian influenza viruses (AIV) are carried subclinically by a variety of waterfowl inhabiting the Chesapeake Bay region [18]. Low pathogenic avian influenza viruses (LPAIV) are regularly detected from both resident and migratory waterfowl in the mid-Atlantic region at a low to moderate frequency [19,20]. While influenza virus transmission among bird populations is largely characterized as direct fecal-oral, the potential for indirect transmission through water and other environmental reservoirs must also be considered [21]. Controlled experimentation has shown that IAV may persist in brackish or salt water at 17 °C for several months [12] and that infectious virus may be recovered from both water and sediment [22]. Influenza virus shed in the feces of waterfowl may thusly persist in the water column for a considerable length of time under appropriate environmental conditions. With considerable overlap in aquatic habitat shared by AIV-susceptible waterfowl and bivalve mollusks in the Chesapeake ecosystem, detection of IAV from tissues of bivalve mollusks from this habitat is not unexpected. It is unknown to what degree bivalves in this region might effectively filter IAV from the water column, what effect accumulation of these viruses by bivalves might have on virus transmission and virulence, and what impacts environmental variables such as seasonality or water quality might have on these interactions. Based on molecular detection of virus alone, we cannot determine whether the IAV material detected in these mollusks represents intact virus or its capability for replication and transmission. We also cannot determine the likely source of the virus, including confirmation that it is of avian origin. Subtype characterization of IAV recovered from bivalves in waterfowl habitat would be an important next step to ascertain the disease ecology related implications of this finding.

Low pathogenic AIV is generally of little consequence to infected waterfowl. Still, it is important to monitor, recognize, and characterize these viruses present in a region such as this one that supports a diversity and high density of susceptible waterfowl and shorebirds as well as a major poultry production industry. Monitoring efforts that utilize sentinel species for the detection of IAV in the environment could enhance biosurveillance programs for these pathogens, and the potential value of bivalve molluscan species as sentinel biosurveillance tools for IAV in the aquatic ecosystem merits further examination. Moreover, the role of filter feeding invertebrate species in the disease ecology of avian influenza in this region remains mostly undescribed and warrants exploration. It is plausible that with further investigation, seasonal variability may be noted in potential IAV recovery from bivalves related to changes in water quality parameters like temperature and salinity, variation in bivalve feeding behavior, and the abundance of waterfowl as primary reservoirs of the virus.

## 4. Materials and Methods

**Figure 1:**
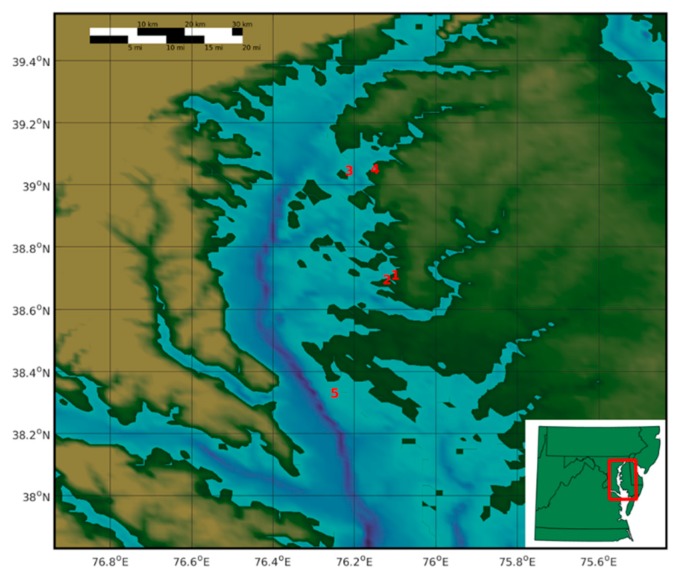
Sampling locations for bivalve mollusk collections in Chesapeake Bay and its tributaries. 1—Trippe Creek; 2—Goldsborough Creek; 3—Eastern Neck; 4—Reed Creek; 5—Barren Island.

Approximately 50 bivalve specimens including clams (*M. balthica*, *Macoma phenax*, *Mulinia* sp., *R. cuneata*, *Mya arenaria*) and mussels (*Guekensia demissa* and an undetermined species) were collected from each of four sampling locations in tidal riverine habitat of the Delmarva Peninsula and one location in the Chesapeake Bay in November 2015 (Figure 1). Two sites, Goldsborough and Trippe Creeks, are tributaries of the Tred Avon River and another, Reed Creek, is a tributary of the Chester River. One site along Eastern Neck Island is located near the mouth of the Chester River. The sites along Eastern Neck and Barren Islands (located in the Chesapeake Bay adjacent to Dorchester County, MD) are part of the Chesapeake Marshlands Wildlife Refuge Complex. Sites were selected based on known proximity to overwintering waterfowl (Anseriformes) and waterfowl observed in or around the habitat as well as suitability as bivalve habitat. All specimens were collected from shallow waters (<1 m depth) and close to shorelines (<20 m). Specimens were recovered from surface sediment (<10 cm depth) except for the *G. demissa* mussel specimens which were collected from pilings along the shoreline with mussels either slightly submerged or in the intertidal zone. After recovery, whole bivalve specimens were identified to species and transported under refrigeration to the laboratory. They were sorted and frozen at −80 °C pending analysis. Species and size of specimens obtained from each site and subsequently processed for analysis are further described in Table 2.

Three hundred tissue samples from bivalve specimens were processed for analysis. One tissue sample per bivalve specimen was collected, processed, and analyzed from the smaller species (*Macoma* spp., *Mulinia* sp., *M. arenaria*, unidentified mussel) and two samples per bivalve specimen were collected, processed and analyzed from the larger species (*Rangia* sp. and *G. demissa*). Tissue samples were handled aseptically and treated individually throughout the entire process as follows using flame-sterilized dissecting instruments. Whole bivalves were thawed slowly on ice, and external surfaces were disinfected with 70% ethanol. Approximately 30 mg of composite tissue (gill, gut, gonad, mantle, muscle) was collected for each sample and placed in sterile bead ruptor tubes (2mm ceramic beads) with 600 mL TRIzol^®^ Reagent (Ambion, Carlsbad, CA). Samples were subjected to bead homogenization on an Omni Bead Ruptor 24 bead mill homogenizer (Omni International, Kennesaw, GA, USA) for three cycles of 40 sec (6 m/s) with intermittent 3 min intervals of samples cooled on ice. Supernatants were collected from the homogenized specimens for RNA extraction via the E.Z.N.A. Viral RNA Kit and Protocol (Omega Bio-tek Inc., Norcross, GA, USA). All extracted nucleic acid samples were stored at −20 °C until analyzed. Sample RNA was transcribed to cDNA using the Applied Biosystems high-capacity cDNA reverse transcription kit (Life Technologies, Carlsbad, CA, USA), using random hexamers, according to the manufacturer’s protocol.

All samples were screened for the presence of IAV via quantitative PCR targeting the influenza A matrix gene [23]. A partial influenza A matrix gene (251 nt) was synthesized using GeneArt^TM^ gene synthesis services (ThermoFisher Scientific, Waltham, MA, USA) for use as a positive control standard. Primers and the synthetic control sequence are listed in Table 3. Influenza type A screening for the matrix gene was performed via qPCR on an ABI viiA™7 real-time PCR system. Reactions were run as 20 µL volumes containing Power SYBR^®^ Green PCR Master Mix (Applied Biosystems, Life Technologies, Carlsbad, CA, USA) and 10 pM of each forward and reverse primer. Cycling parameters were 50 °C for 2 min and 95 °C for 10 min followed by 40 cycles of 95 °C for 10 s, 58 °C for 15 s and 72 °C for 20 s. An appropriately sized amplification product was confirmed for each reaction by electrophoresis of 5 µL of the reaction product through a 1.2% I.D.NA^™^ agarose gel (Cambrex Corporation, East Rutherford, NJ, USA) at 100 V for 45 min.

## Figures and Tables

**Table 1:** Bivalve specimens testing positive for the influenza A virus matrix gene by RT qPCR methodology.

Site Designation	Species	# Specimens Tested	# Positive Specimens	% Positive Specimens
Trippe Creek	*Macoma balthica*	59	2	3.4
Goldsborough Creek	*Macoma phenax*	40	4	10.0
Reed Creek	*Guekensia demissa*	24	6	25.0

#: Number of specimens tested.

**Table 2:** Bivalve species sampled from Chesapeake Bay and tributaries, November 2015.

Site Designation	Approx. Latitude	Approx. Longitude	Species (# Specimens)	Mean Weight (mg)	Mean Length (mm)
Trippe Creek	38.710299	−76.110816	*Macoma balthica* (59)	1180	19
			*Macoma phenax* (16)	155	9
Goldsborough Creek	38.694905	−76.131430	*Macoma balthica* (10)	148	10
			*Macoma phenax* (40)	100	8
			*Mulinia* sp. (7)	287	10
Eastern Neck	39.045982	−76.223874	*Macoma balthica* (48)	1310	21
			*Macoma phenax* (2)	130	10
			*Rangia* sp. (11)	23,345	40
			*Mya* sp. (1)	2800	32
			Mussel undet sp (1)	160	11
Reed Creek	39.050508	−76.160033	*Guekensia demissa* (24)	6691	42
			*Rangia* sp. (1)	19,100	37
Barren Island	38.333101	−76.259680	*Macoma balthica* (36)	210	11
			*Mulinia* sp. (8)	213	9

#: Number.

**Table 3:** Primers and synthetic standard used for screening the type A matrix gene by qPCR.

Description	Sequence
Forward primer (M+25)	AGATGAGTACTTCTAACCGAGGTCG
Reverse primer (M-124)	TGCAAAAACATCTTCAAGTCTCTG
Synthetic Matrix Standard	TGAAAGATGAGTCTTCTAACCGAGGTCGAAACGTACGTTCTCTCTATCGTCCCGTCAGGCCCCCTCAAAGCCGAGATCGCGCAGAGACTTGAAGATGTTTTTGCAGGGAAGAACACCGATCTCGAGGCACTCATGGAATGGCTAAAGACAAGACCAATCCTGTCACCTCTGACTAAGGGGATTTTAGGATTTGTGTTCACGCTCACCGTGCCCAGTGAGCGAGGACTGCAGCGTAGACGCTTTGTCCAGAA
